# Nanoencapsulation of sophorolipids in PEGylated poly(lactide-co-glycolide) as a novel approach to target colon carcinoma in the murine model

**DOI:** 10.1007/s13346-020-00750-3

**Published:** 2020-04-01

**Authors:** Yusuf Haggag, Mohamed Elshikh, Mohamed El-Tanani, Ibrahim M Bannat, Paul McCarron, Murtaza M. Tambuwala

**Affiliations:** 1 Department of Pharmaceutical Technology, Faculty of Pharmacy, Tanta University, Tanta, Egypt; 2 School of Biomedical Sciences, Ulster University, Cromore Road, Coleraine, Co. Londonderry BT52 1SA UK; 3 Pharmacological and Diagnostic Research Centre, Al-Ahliyya Amman University, Faculty of Pharmacy, Amman, Jordan; 4 School of Pharmacy and Pharmaceutical Sciences, Ulster University, Cromore Road, Coleraine, Co. Londonderry BT52 1SA UK

**Keywords:** Sophorolipids, Biosurfactant, Drug delivery, Nanocapsule, CT26 cells, Colon cancer

## Abstract

Poly(lactic-co-glycolic acid) nanocapsules containing amphiphilic biosurfactant sophorolipids were formulated using a dispersion-based procedure. Di-block copolymers were used to vary peripheral poly(ethylene glycol) density, and variation in the oil core was used to achieve efficient encapsulation of the sophorolipid payload. Particulate size, zeta potential, encapsulation efficiency, release and stability were characterised. A glyceryl monocaprate core composition had the lowest particulate size, maximum encapsulation efficiency and optimum shelf-life stability compared to other formulations. This core composition was used to deliver sophorolipid to both in vitro and in vivo model tumour cell lines (CT26 murine colon carcinoma) and the effect of peripheral hydrophilicity was evaluated. Formulations with 10% poly(ethylene glycol) density achieved more than 80% reduction in cancer cell viability after 72 h and enhanced cellular uptake in CT26 cells. These formulations exhibited higher tumour accumulation and a longer blood circulation profile when compared to the non-poly(ethylene glycol)-containing nanocapsules. Animals treated with sophorolipid-loaded nanocapsules showed a tumour growth inhibition of 57% when compared to controls. An assessment of tumour mass within the same study cohort showed the biggest reduction when compared control and free drug-treated cohorts. This study shows that hydrophilic poly(lactic-co-glycolic acid) nanocapsules loaded with sophorolipids can address the poor intracellular delivery associated with these biosurfactants and is a promising approach for the treatment of colon neoplasia.

**Figure:**
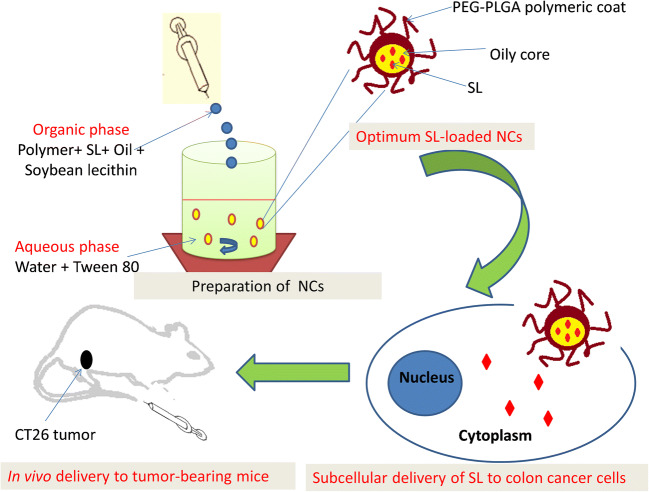
Graphical abstract

Graphical abstract

## Introduction

Natural surfactants are classified according to their chemical composition and source of origin (plant or microorganism) [1]. Specific types derived from microorganism-based sources are characterised further on the basis of molecular weight and segregated into two separate classes, known as the biosurfactants and the bioemulsifiers. The former possess excellent surface activity and are able to lower interfacial tensions between immiscible phases. However, the bioemulsifiers are less effective at doing this, yet still act as effective emulsifiers [2]. These useful properties give rise to a broad range of applications spanning the pharmaceutical and medicinal fields. These include inhibition of bacterial growth, stimulation of immune response, enzyme stimulation, tumour growth inhibition, cell lysis (haemolysis), food digestion and antifungal activity [3].

Biosurfactants with a carbohydrate moiety (glycolipid) have therapeutic potential in the management of disease. Four main types are recognised. These are the (i) sophorolipids (SL), (ii) trehalolipids, (iii) mannosylerythritol lipids and (iv) rhamnolipids [4]. The SL biosurfactants are of particular interest and produced by yeasts, such as *Candida floricola*, *Candida apicola*, *Candida bastistaeic* and *Starmerella bombicola* [5]. SL are assembled using a hydrophilic carbohydrate head, composed of a disaccharide (sophorose), linked to a hydrophobic fatty acid tail [6]. Recently, the anticancer activity and tumour growth inhibition of SL in different types of cancer cell lines have been reported [7, 8]. Activity has been demonstrated against a broad range of tumour-derived cell lines, including liver (H7402 cells), lung (A549 cells) and leukaemia (K562 cells) [5]. Further reports also show activity against breast (MDA-MB231 cells) [9] and colon (HT29, HT115, HCT116 cells) [10]. A detailed biomolecular study performed on HeLa cells demonstrates that lactonic sophorolipids induce features associated with apoptosis, along with expression of pro-apoptotic biomarkers, such as caspase-3 and caspase-12 and activation through endoplasmic reticulum signalling pathways with cell-cycle blocking at both G_0_ and G_2_ phases [11].

Although preliminary investigations demonstrate promise, the translational applications of SL for the treatment of disease have been hindered by a number of factors, such as high production cost, limited purification, poor aqueous solubility and an overall lack of understanding of interactions with cells [1]. Therefore, an aim of this present study was to evaluate a nanoparticulate-based delivery strategy to enhance administration of SL and address the poor cellular bioavailability. Given that colloidal carriers target specified locations and overcome issues associated with poor drug solubility [12, 13], the design approach chosen was based on polymeric nanocapsules (NCs). They exhibit a distinctive core-shell structure in which active water-insoluble drug substances are confined in an oil core surrounded by a polymeric membrane [14]. In addition, encapsulation enables sustained and controlled release kinetics in both in vitro and in vivo systems [15–17]. These beneficial attributes may overcome the problems associated with delivery of SL due to its poor water solubility.

A second aim of the study was to evaluate the effect of polymer hydrophilicity. Previous studies have shown that NC formulated from PEGylated PLGA polymers have improved the anticancer effects of lipophilic drugs, such as curcumin, used in the investigational treatment of colon neoplasia [12, 18]. PEGylated PLGA was used as it has been shown to be an effective encapsulating matrix with reported enhancement of the antitumour activity of various anticancer molecules, such as small molecular chemotherapeutics or larger biomolecular anticancer peptides [19–21]. Furthermore, colloidal PEGylated PLGA formulations possess (i) high drug loading of both hydrophilic and hydrophobic drugs, (ii) controlled release profiles, (iii) good in vitro stability and (iv) novel bio-distribution arising from passive targeting [22, 23]. The third aim was to evaluate cellular uptake, localisation and cytotoxicity of SL-loaded NC using a colon cancer cell and an in vivo murine model. To the best of our knowledge, this is the first study to have demonstrated the antitumour activity of SL-loaded NC against CT-26 colon cancer cells (in vitro*)* and in in vivo murine model.

## Materials and methods

### Materials and reagents

PEG-PLGA diblock copolymers (Resomer® RGP d 5055 (5% PEG of MW 5 kDa) and Resomer® RGP d 50105 (10% PEG of MW 5 kDa)) were purchased from Boehringer Ingelheim Ltd. (Ingelheim, Germany). PLGA with a 50:50 lactic:glycolic ratio (Resomer® RG 503H, MW 34 kDa) was purchased from Sigma-Aldrich (St. Louis, USA). Sophorolipid (1′,4″-Sophorolactone 6′,6″-diacetate; from yeast), coumarin 6 (3-(2-Benzothiazolyl)-7-(diethylamino)coumarin, 3-(2-Benzothiazolyl)-N,N diethylumbelliferylamine), DiR (1,1′-dioctadecyl-3,3,3′,3′-tetramethylindotricarbocyanine iodide), DAPI (4′,6-diamidino-2-phenylindole), dialysis tubing (MWCO 2000 Da), SP-Sephadex C-25 resin, castor oil, palm oil, Tween® 80, acetone and absolute ethanol (ultra-pure grades) were obtained from Sigma-Aldrich (St. Louis, USA). Glyceryl monocaprate (Capmul® MCM C10) was a generous gift from ABITEC Corporation, USA. RPMI-1640 and DMEM media, trypsin/EDTA, phosphate buffered saline (PBS), fetal bovine serum (FBS) and penicillin/streptomycin were obtained from Gibco, Invitrogen, UK. Isoflurane (IsoFlo®) was purchased from Abbott Laboratories Ltd. (UK).

### Solubility of SL in the oil phase

The solubility of SL in three different oils used for NC preparation (castor oil, palm oil and glyceryl monocaprate) was determined using a simple saturation method [12]. An excess of SL (5 mg) was placed in glass vials containing a sample of the test oil (1.0 ml) and agitated at 100 rpm for 48 h at 37 °C. After centrifugation, 100 μl of supernatant was withdrawn and mixed with an acetone/ethanol mixture (1:1 v/v). The concentration of SL dissolved in the organic solvent was determined using a calibrated UV spectrophotometric method, measuring absorbance at 207 nm (Model UV-1601 PC; Shimadzu, Kyoto, Japan) [24].

### Preparation of SL-loaded polymeric NC

SL-loaded NCs were prepared using a modified nanoprecipitation method [25]. Polymer (50 mg), SL (5 mg), soybean lecithin (25 mg) and oil phase (300 μl) were dissolved in 5 ml of an acetone/ethanol (1:1 v/v) mixture. This non-aqueous phase was added dropwise into an aqueous phase (10 ml) containing 0.2% w/w of Tween® 80. The final mixture was stirred for 30 min to facilitate solvent diffusion and formation of NC. Organic solvent was eliminated using evaporation under reduced pressure and rotation. Blank NC (non-SL-loaded) were prepared using identical conditions but without adding SL to the oil phase. The SL-loaded NC suspension was purified by passing down a PD10 desalting column (GE Healthcare) installed on a purifier system with an eluent composition of 50 mM potassium phosphate and 100 mM NaCl (pH 7) and a flow rate of 1.0 ml min^−1^ to remove non-entrapped drug.

Fluorescent SL-loaded NCs were prepared by adding either coumarin 6 (100 μg) or DiR (150 μg) to the non-aqueous phase. Coumarin 6 was chosen for qualitative cellular uptake analysis, and DiR labelling was used an instrument-specific dye to provide optimum in vivo biodistribution imaging data. Process variables, such as the polymer type and composition of the oil core, were modified, as shown in Table 1, along with the identifying code for each SL-loaded NC formulation.

**Table 1:** Formulation identifiers for polymer type and oil core

Formulation identifier	Polymer type	Oil core
F1	PLGA	Castor oil
F2	PLGA	Palm oil
F3	PLGA	Glyceryl monocaprate
F4	5% PEG-PLGA	Castor oil
F5	5% PEG-PLGA	Palm oil
F6	5% PEG-PLGA	Glyceryl monocaprate
F7	10% PEG-PLGA	Castor oil
F8	10% PEG-PLGA	Palm oil
F9	10% PEG-PLGA	Glyceryl monocaprate

### Physicochemical characterisation of SL-loaded NC

Particle size, size distribution and poly dispersity index (PDI) of SL-loaded NCs were determined using dynamic light scattering at 90° (Zetasizer 5000, Malvern Instruments, Malvern, UK). A liquid aliquot from the SL-loaded NC colloidal suspension was diluted in ultrapure water and an average of three measurements was recorded. Zeta potential (ζ-potential) of SL-loaded NC was determined using laser Doppler electrophoresis applied to an aliquot of colloidal suspension, diluted in aqueous 0.001 M KCl to adjust for conductivity (Zetasizer 5000, Malvern Instruments, Malvern, UK). Morphology was examined using transmission electron microscopy (TEM) (JOEL JEM 2000 EX200). A sample of NC suspension was placed on a Formvar-coated grid operating at an accelerating voltage of 80 kV. Fluorescent SL-loaded NCs were evaluated using similar methods for particle size, polydispersity index and zeta potential.

### In vitro stability

Samples of SL-loaded NC suspensions were sealed in glass vials and stored in the dark under refrigeration (4 °C). Stability was evaluated every 10 days by visual inspection of physical properties (colour, turbidity and opacity) and by determination of size, PDI and zeta-potential, as described in “Physicochemical characterisation of SL-loaded NC” section. Each sample was tested three times and the measurements were presented as an average ± SD.

### Determination of SL encapsulation efficiency (%EE)

The percentage SL encapsulation efficiency (%EE) in SL-loaded NC was determined by an indirect method of analysis. The amount of non-encapsulated SL in fractions following column purification of a NC suspension was determined by HPLC (Shimadzu, Tokyo, Japan). A reversed-phase column (Phenomenex-Luna® C18–5 column mm, 5 μm) and a mobile phase, at a flow rate of 1.0 ml min^−1^, comprising a linear gradient of acetonitrile and water over a run time of 40 min, were used [24].

### In vitro release profile

A liquid sample of SL-loaded NC formulation containing a predetermined amount of SL was placed into a 10-kDa dialysis bag. The dialysis membrane was dialysed against a receiver medium of 50 ml PBS containing Tween® 80 (1% w/v) at pH 7.4. The release was studied at 37 °C at 100 rpm. At predetermined time interval points, 1.0 ml sample was taken and replaced by an equivalent amount of fresh medium, thereby maintaining sink conditions. Drug concentration was assessed by HPLC, as previously described.

### Seeding of CT26 and CCD-841-CoN cells

CT26 murine colon carcinoma (CT26) cells were grown in 100 cm^2^ canted-neck tissue culture flasks. Cells were cultured in complete RPMI media supplemented with FBS (10% v/v), L-glutamine (2 mmol l^−1^), penicillin (100 U ml^−1^) and streptomycin (100 μg ml^−1^). CCD-841-CoN, normal colonic epithelium cells, were maintained in complete DMEM media supplemented with 10% fetal bovine serum. Cells were passaged every 3 days at 80% confluence. All cultures were incubated at 5% CO_2_ and 37 °C.

CT26 cells were harvested when the cells were 90% confluent. The cell suspension was centrifuged at 1500*g* (4 °C) for 5 min and the pellet resuspended in RPMI complete growth medium. A cell count was performed on a sample of cell suspension (10 μl) using a haemocytometer. CCD-841-CoN cells were seeded using similar conditions, with DMEM complete growth medium being used instead of RPMI medium.

### Fluorescence analysis of SL-loaded NC cellular uptake

Cellular uptake of coumarin 6-tagged SL-loaded NC (F3, F6 and F9) was evaluated using flow cytometry and fluorescence microscopy [20]. Briefly, for flow cytometric analysis, coumarin 6-labelled NCs were suspended in Optimem® media and added to CT26 cells for 24 h in 6-well plates. Cells were collected by trypsinisation and treated with FACS buffer. Cellular uptake of SL-loaded NC was quantified by gating cells for positive coumarin 6 fluorescence for CT26 control cells (untreated) and cells treated with either free coumarin 6 or coumarin 6-labelled NC in the FITC channel detector (BD FACS Calibur flow cytometer, BD Biosciences). The measurements were performed in triplicate.

Cellular localisation of coumarin 6-tagged SL-loaded NC was evaluated qualitatively by fluorescence microscopy (Olympus DP-71, Olympus, Center Valley, PA, USA). Intracellular distribution of fluorescent NC into CT26 cells was detected using fluorescence imaging 1 day after treatment. CT26 cells were seeded into a 6-well plate, containing two fixed coverslips incubated into 2.0 ml of RPMI growth medium. Coverslips were mounted using mounting media (VectaShield) and subsequently counterstained with DAPI. Cellular localisation of labelled NC was represented by the green signals, while cells nuclei were detected in blue. The untreated CT26 cells and cells treated with free coumarin 6 were used as negative and positive controls, respectively.

### In vitro cell viability assay

CT26 murine colon cancer cells and CCD-841-CoN normal colon epithelium cells were seeded in 24-well plates and incubated with 0.5 ml of complete growth media. After 24 h, cells were treated with blank NC or three different concentrations of SL (20, 40 and 60 μM) in either the free form or the equivalent amount of SL-loaded NC (F9). Cytotoxicity was examined using a modified MTT assay [26]. After treatment for 24 h, the media were removed and 500 μl of MTT solution (15% v/v) was added to the cells. They were incubated for 3 h at 37 °C and 5% CO_2_. DMSO (500 μl) was added, and the absorbance at 570 nm was recorded (FLUO Star Omega microplate reader, BMG Labtech). Results were expressed as the percentage cell viability, compared to controls. Each treatment was tested three times and the measurements were presented as an average ± SD (*n =* 3).

### In vivo studies

Female Balb/c mice, weighing approximately 20 g and aged between 4 and 6 weeks, were allowed to acclimatise to laboratory conditions for 10 days before starting the experiment. Mice were fed water and standard pellet chow ad libitum for the duration of the in vivo experiment. All animal experiments were performed in compliance with the UK Home Office (1989) Code of Practice for the Housing and Care of Animals used in Scientific Procedures. A xenograft model of CT26 colon carcinoma was induced by implanting 1 × 10^6^ cells in 0.1 ml PBS subcutaneously at both lower flanks [12]. A palpable solid tumour mass developed 2 weeks following subcutaneous inoculation. All mice rendered tumour-bearing were included in this study. The treatment protocol was started on day 14 and extended to day 30 post-implantation.

Three different groups (*n = 4*) were used in an organ biodistribution study. Control animals in the first group were injected with PBS. The second group was treated with F3 and the third group was treated with F9 through IV injection. The treated groups were injected intravenously with prepared DiR-labelled SL-loaded NC in PBS solution at a dose of 10 mg SL kg^−1^ and scanned using an in vivo imaging system (IVIS Lumina Series III, Caliper Life Sciences, Perkin Elmer, USA) at 1-, 4- and 24-h post-injection [12]. All animals were kept anaesthetised with 1.5% isoflurane/98.5% oxygen to maintain sedation during the scanning process. Ex vivo imaging for excised major organs (heart, lung, liver, spleen, kidney and tumours) was carried out immediately after full in vivo scan. Fluorescence images were obtained using a DiR filter with an exposure time of 1 s. All images were captured using sequential acquisition spectra under un-mixing mode. The collected fluorescence emission signals were measured as mean fluorescence intensity.

Four groups (*n =* 6) were used in an in vivo antitumour activity study. Once anaesthetised, subjects were injected via the tail vein with the following:

- (Group 1) 200 μL of saline (control group);
- (Group 2) 200 μL of blank NC nano-suspension;
- (Group 3) 200 μL free SL at a dose of 10 mg kg^−1^;
- (Group 4) 200 μL of SL-loaded NC (F9) nano-suspension containing SL at a dose of 10 mg kg^−1^.

All treatments were injected every 3 days with a total of 5 injections during the whole experiment period. Tumour volumes were recorded at the beginning of the treatment at day 14 post-implantation and every 2 days, thereafter, until the end of the experiment. A digital calliper was used to record dimensions (mm) and the whole tumour volume was recorded. Mice were sacrificed after the end of the experiment by cervical dislocation, and the tumour was excised and washed with saline to measure its final mass.

### Statistical analysis

For all in vitro experiments, data were presented as mean ± SD. In in vivo experiment, results were presented as mean ± SEM. Significant differences between different groups were examined using one-way ANOVA followed by post hoc Tukey’s test. *p <* 0.05 was considered statistically significant in all experiments.

## Results and discussion

Polymeric SL-loaded NCs were fabricated using the nanoprecipitation method, and the impact of formulation variables, such as PEG density on the PLGA chain and the type of oil core, on the physicochemical characteristics of the SL-loaded NCs was investigated. Mean particle size, PDI, zeta potential, encapsulation efficiency, in vitro release profile and in vitro stability were determined. The aim of the work was to use this data and select an optimised formulation for further in vivo evaluation. This optimised formulation was considered to be one with the smallest diameter, best encapsulation efficiency, longest shelf-life stability and controlled therapeutic action.

### Effect of polymer type on physicochemical properties

**Fig. 1:**
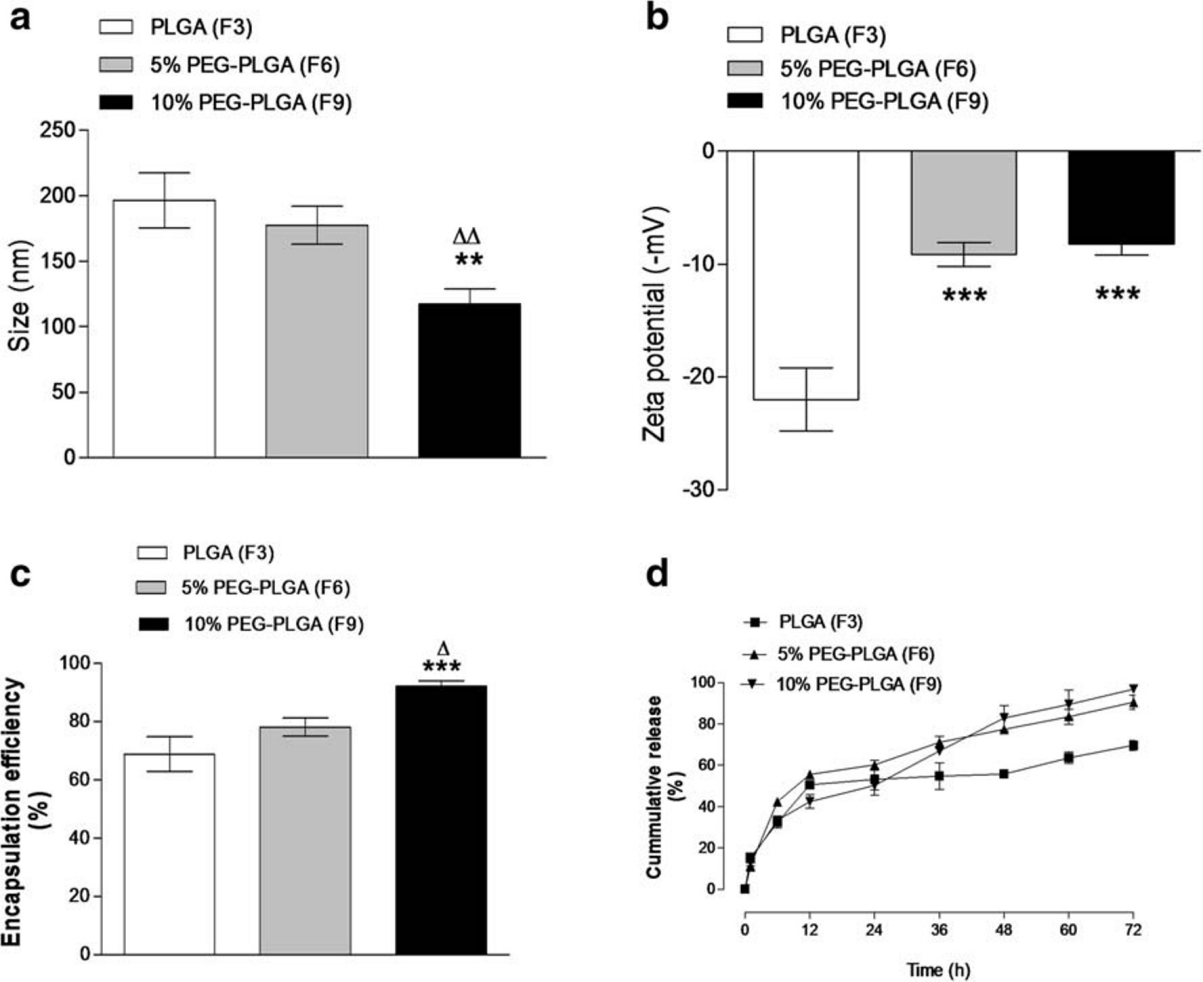
Effects of polymer type on NC size (**a**), zeta potential (**b**), encapsulation efficiency (**c**) and SL in vitro release (**d**). Values are mean ± SD with *n =* 3. For 1**a**–**c**, *p* *<* 0.05, **p < 0.01, ***p < 0.001 compared with PLGA (F3). ^Δ^*p <* 0.05, ^ΔΔ^*p <* 0.01 compared with 5% PEG-PLGA (F6)

Modification of PLGA by incorporation of PEG blocks within the polymeric structure is a common experimental variable within the formulation strategy of a nanoparticulate system. Effects on aqueous solubility of poorly soluble payloads, aggregation, stability and opsonisation are often reported [12, 23]. However, the approach is also associated with further advantages, such as improvement in antitumour efficiency and targeting characteristics [19, 20]. Therefore, the physicochemical properties of different SL-loaded NC (F3, F6 and F9) fabricated from three different PLGA polymers were investigated (Fig. 1). PLGA-based NC (F3) (196.5 ± 20.9 nm) were significantly larger in size (*p <* 0.01) than NC prepared from 10% PEGylated polymers of (F9) (117.5 ± 11.5 nm). A non-significant difference was observed when compared to 5% PEGylated NC (F6) (177.4 ± 14.5 nm) (*p ˃* 0.05). SL-loaded NC prepared from 10% PEG-PLGA diblock copolymers (F9) were significantly smaller than NC prepared from 5% PEG-PLGA copolymer (*p <* 0.01), (Fig. 1a). Similar results were observed during the preparation of polymeric nanospheres using the same polymers [22, 23]. This was attributed to the effect of linked PEG molecules on the assembly of smaller NC. Modification of PLGA backbone by addition of PEG as a hydrophilic polar segment can affect the physicochemical properties of the SL-loaded NCs prepared from PEGylated copolymers. The particle size is often increased by an increase in the hydrophobic segment of the used copolymer. Higher PEG content resulted in a smaller NP size, attributable to the short chain length of 10% PEG-PLGA copolymer compared to 5% PEG-PLGA and PLGA [23, 27]. All SL-loaded NC showed low polydispersity index ranging from 0.19 to 0.38. Increasing the PEG content in the NC polymeric coat resulted in a significant increase in PDI values compared to PLGA NC (*p <* 0.05).

The *ζ*-potential of NC colloidal suspension plays an important role in maintaining in vitro stability. In vitro stability of colloidal suspension depends on the degree of electrostatic repulsion between nanoparticles. Higher negative or positive zeta potential values resulted in good in vitro stability with low tendency of particles aggregation [26]. F3 exhibited higher negative *ζ*-potential values (− 21.9 ± 2.8 mV) compared to the PEGylated PLGA NC (F6 and F9) (*p <* 0.001) (Fig. 1b). Increasing the PEG content in the backbone of the polymeric coat from 5 to 10% had a non-significant effect on the *ζ*-potential (*p ˃* 0.05). The lower negative *ζ*-potential of PEGylated NC is due to the presence of surface-decorated PEG chains which mask the free negative surface charge represented by the free anionic groups of PLGA [22, 23].

The presence of PEG blocks in the copolymer structure resulted in a significant increase in SL entrapment (*p <* 0.001). Encapsulation efficiency was increased from 68.8 ± 6 in PLGA to 92.18% ± 1.8% in 10% PEG-PLGA (Fig. 1c). This can be explained by the amphiphilic nature of both the polymer itself and SL. PEG regions of the copolymer will most likely orientate towards the continuous aqueous phase, which can associate with the hydrophilic regions of the SL molecule. The hydrophobic part of the SL molecule will orientate towards the oil core. Therefore, the PEG-PLGA diblock copolymer creates a suitable microenvironment created by its amphiphilic nature, leading to high SL loading [28].

The in vitro release profile showed comparable burst release of SL from the three different polymers (*p ˃* 0.05). Burst release after 24 h was 53.2% ± 8.9, 60% ± 4.2 and 50% ± 8.3 from F3, F6 and F9, respectively (Fig. 1d). The observed burst release can be attributed to the high amount of SL attached to the surface of PLGA and PEGylated due to its amphiphilic nature and surface-active properties. Release of SL from PLGA NC (F3) was sustained after the first 24 h, and release became slower, with approximately 70% of SL released over the experimental period of 72 h. The PEGylated PLGA NC showed the faster release of SL after the first 24 h with approximately 90% and 97% of SL released over 72 h from F6 and F9, respectively.

### Effect of oil core type

**Fig. 2:**
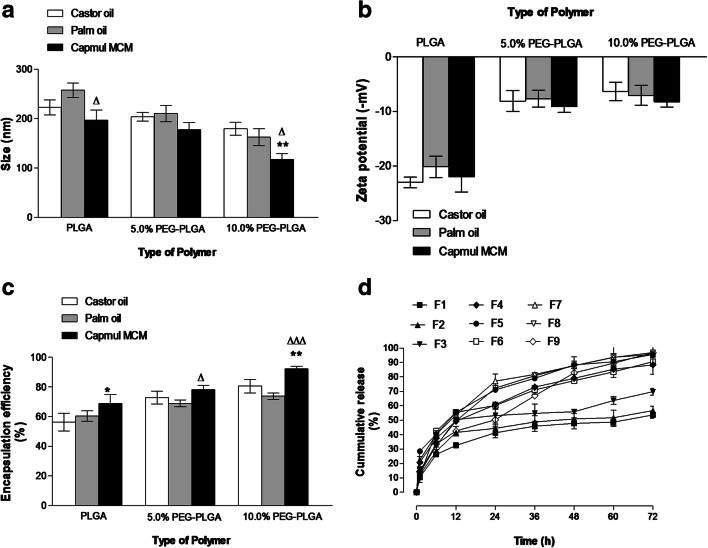
Effects of the oil core type on NC size (**a**), zeta potential (**b**), encapsulation efficiency (**c**) and in vitro SL release (**d**). Values are mean ± SD with *n =* 3. For 2**a**–**c**, *p < 0.05, **p < 0.01, *p <* 0.001 compared with castor oil for each polymer type. ^Δ^*p <* 0.05, *p <* 0.01, ^ΔΔΔ^*p <* 0.001 compared with palm oil for each polymer type

SL was poorly soluble in palm oil (1.06 mg ml^−1^), being significantly lower than that in both castor oil (1.79 mg ml^−1^) and glyceryl monocaprate (2.21 mg ml^−1^). The physicochemical characteristics of different SL-loaded NC fabricated using these different oil cores are shown in Fig. 2. SL-loaded NC based on glyceryl monocaprate (F9) exhibited significantly lower size (117.5 ± 11.5 nm) compared to NC-palm oil (F8) (162.5 ± 17.2 nm) and NC-castor oil (F7) (179.2 ± 13.1 nm) for the 10% PEGylated copolymer types (*p <* 0.05). However, this trend was not apparent in the PLGA NC (Fig. 2a). Type of oily core had a significant impact on the physicochemical properties of SL-loaded NCs. At room temperature, vegetable oils such as palm oil and castor oil showed higher viscosity relative to glyceryl monocaprate. The smaller size (*p* < 0.05) observed for (F9) is probably a consequence of the lower viscosity of the glyceryl monocaprate compared with the palm oil and castor oil used for preparation of F8 and F7, respectively [29]. The PDI values (data not shown) for glyceryl monocaprate cores were significantly lower than the palm oil and castor oil formulations. Variation in oil type did not have any significant effect on the *ζ*-potential of the different NC formulations (*p ˃* 0.05) (Fig. 2b).

The % encapsulation efficiency of all SL-loaded NC ranged from 56.3 ± 5.9 to 92.18% ± 1.8%. Glyceryl monocaprate-based NC exhibited the highest encapsulation efficiency values compared to other oil-based formulation, regardless the type of the polymer (Fig. 2c). This is attributed to solubility of SL in glyceryl monocaprate, which prevented its undesirable loss towards the aqueous phase leading to a high degree of entrapment. In addition, glyceryl monocaprate has a lower hydrophilic–lipophilic balance (HLB approximately 5–6) than castor oil (HLB 14) and palm oil (HLB 10). Therefore, NC based on a glyceryl monocaprate core may offer a favourable microenvironment for encapsulating water-insoluble glycolipids, such as lactonic SL [30]. This finding has been demonstrated in other work, where the highest encapsulation efficiency of paclitaxel was observed in glyceryl monocaprate nanocapsule cores compared with that in other oils (palm oil and coconut oil) [31]. Similar findings are observed for entrapment of curcumin into castor oil-based nanocapsule due to enhanced solubility in the core medium [12].

In vitro release profiles showed that changing the oil core influenced the initial burst release from different NC (Fig. 2d). In PLGA-based NC, a higher burst release had been observed from F3 (containing glyceryl monocaprate) compared to F1 and F2 containing castor oil or palm oil, respectively. The opposite effect was then observed for PEGylated NC under the same conditions. The initial burst release after 24 h was less for glyceryl monocaprate compared to other oils. This might be attributed to a polymer-oil interaction driven by the relative hydrophilicity of the polymer. The higher burst release for PLGA NC might be due to its favourable association with glyceryl monocaprate, which led to longer solidification time and, consequently, to the formation of more porous NC structure, facilitating drug release [20].

### Transmission electron microscopy

**Fig. 3:**
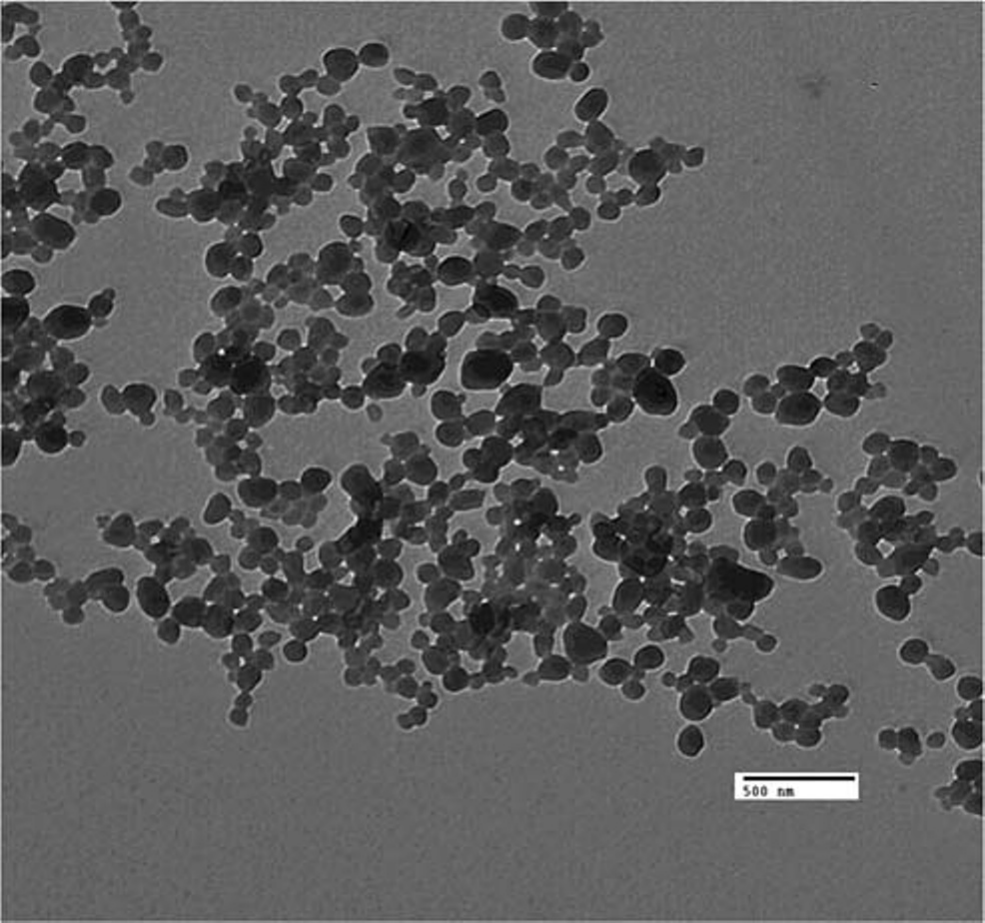
TEM images of SL-loaded NC (F9) after formulation

The appropriate morphology of nanoparticulate drug delivery system is crucial for the successful design of therapeutic drug delivery systems. In this work, TEM was used to visualise the particulate and to evaluate aggregation or adhesion of SL-loaded NC. TEM images revealed a smooth spherical shape of (F9) homogenous size with no aggregations (Fig. 3). The average size obtained from TEM was comparable to what obtained by light scattering.

### In vitro stability of SL-loaded NC

After formulation of all SL-loaded NC, non-entrapped drug was removed by gel filtration chromatography. The purified NC formulations (F3, F6 and F9) were selected for further study as they showed superior physicochemical properties for each polymer type compared to other NC. These formulations were characterised for their size, PDI, *ζ*-potential immediately after purification at time zero and every 10 days over 1 month of storage at 4 °C (Table 2). The results from assessment of stability parameters, such as time-dependent variation in particle size, confirmed that F3 and F9 were sufficiently stable for 1 month after preparation. There was a non-significant (*p ˃* 0.05) increase of size, PDI and zeta potential compared to freshly prepared ones. Although F6 prepared from 5% PEG-PLGA diblock copolymer showed non-significant (*p ˃* 0.05) increase in size and zeta potential, it showed a significant (*p ˂* 0.05) increase in PDI value after 30 days from preparation. No apparent change in colour, clarity or phase separation was observed in any of NC preparations. Herein, stability results showed that SL-loaded NCs prepared from 10% PEG-PLGA copolymer were biocompatible, stable and monodisperse nanosystem for SL encapsulation, with potential applications for drug delivery. Our results prove that nanoprecipitation technique leads to efficient stabilisation of oily core PEGylated NCs. The in vitro stability measurements showed sufficient colloidal stability for 1 month. Stability results confirmed lack of evident destabilisation phenomena associated with no degradation over time which can affect the size, PDI and zeta potential of the prepared NCs.

**Table 2:** In vitro stability SL-loaded NC

Formulation identifier	Time (day)	Size (nm)	PDI	*ζ*-potential (mV)
F3	0	196.5 ± 20.9	0.19 ± 0.03	− 21.9 ± 2.8
	10	205.3 ± 18.7	0.18 ± 0.04	− 19.5 ± 2.5
	20	217.7 ± 21.5	0.22 ± 0.05	− 18.7 ± 3.9
	30	226.4 ± 15.3	0.25 ± 0.03	− 19.8 ± 3.4
F6	0	177.4 ± 14.5	0.26 ± 0.02	− 9.1 ± 1.01
	10	191.5 ± 11.9	0.29 ± 0.05	− 10.2 ± 2.1
	20	209.3 ± 16.8	0.28 ± 0.04	− 8.9 ± 2.4
	30	217.3 ± 19.5	0.35 ± 0.03⃰	− 7.9 ± 1.6
F9	0	117.5 ± 11.5	0.29 ± 0.04	− 8.2 ± 0.97
	10	126.7 ± 13.6	0.3 ± 0.07	− 7.7 ± 1.2
	20	135.3 ± 15.8	0.33 ± 0.03	− 8.1 ± 2.4
	30	143.3 ± 12.2	0.35 ± 0.04	− 7.8 ± 1.9

Results are presented as mean ± SD with *n =* 3

*p ˂ 0.05 compared to day 0 for the same formula

### Cellular uptake of SL-loaded NC

**Fig. 4:**
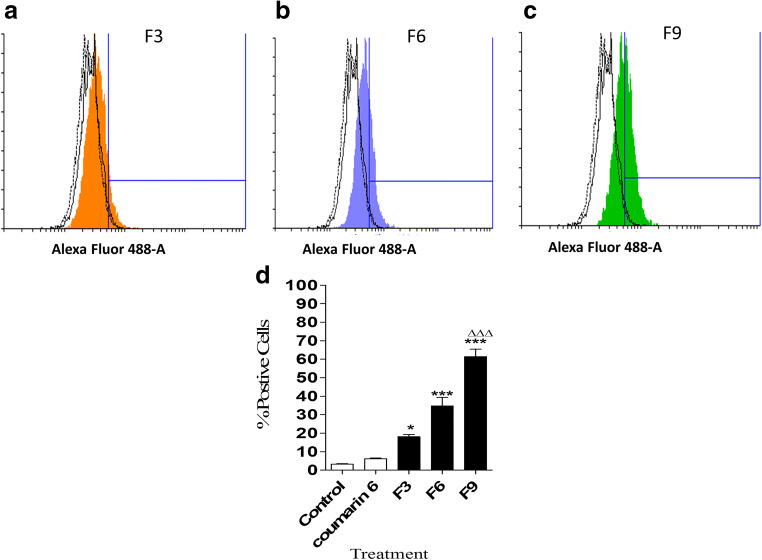
Quantitative cellular uptake of different types of SL-loaded NC after 24 h, as determined by flow cytometry. Individual histograms representing fluorescence shift resulted after treatment with F3 (**a**), F6 (**b**) and F9 (**c**). Direct comparison of percentage of positive cells showing cellular uptake of each NC (**d**). Values are mean ± SD with *n =* 3. For 4**d** ***p < 0.001 compared with control and coumarin 6 groups. ^ΔΔΔ^*p <* 0.001 compared with F3 and F6

**Fig. 5:**
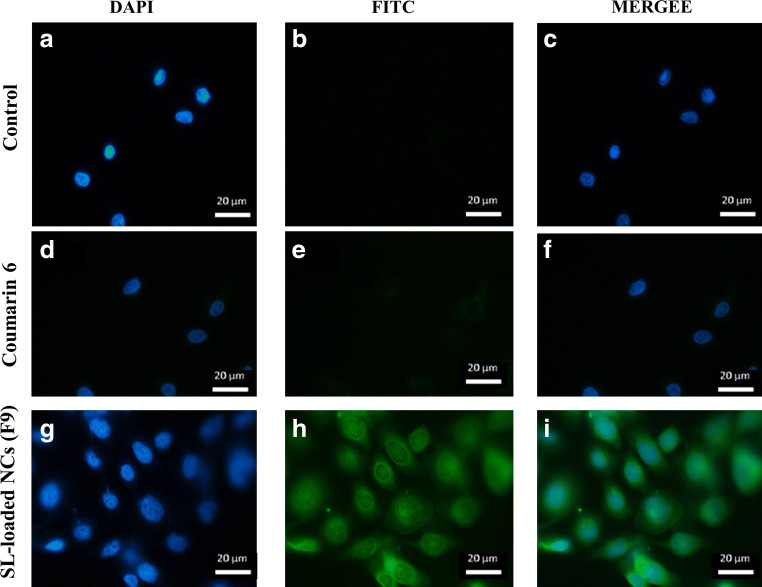
Fluorescence microscope images of control cells (**a**–**c**), cells treated with coumarin 6 (**d**–**f**) and coumarin 6 tagged SL-loaded NP (F9) (**g**–**i**) after 24 h of treatment

This study investigated the efficiency of cellular uptake of SL-loaded PLGA and PEGylated PLGA NC by CT26 colon cancer cells. The effect of polymer type on the in vitro cellular uptake results are shown in Figs. 4 and 5. The quantitative flow cytometry analysis was used to detect the uptake of the coumarin 6-tagged SL-loaded NC (F3, F6 and F9) in CT26 colon cancer cells after 24 h of treatment (Fig. 4a–d). The intracellular uptake of fluorescent NC into CT26 cells was expressed by a fluorescence shift from control cells depending on the fluorescence intensity (Fig. 4a–c). The extent of cellular uptake was measured secondly by the percentage of cells with positive staining following treatment (Fig. 4d). After 24 h, F9 exhibited significantly higher cellular uptake (*p <* 0.001) than other NC formulations with lesser amounts of PEG in the polymer coat (F3 and F6). Interestingly, these results were in good agreement with the optimum physio-chemical properties of F9, with the lowest particle size, highest PEG content and lowest zeta potential [20]. These parameters gave rise to the greatest uptake in CT26 cells. Previous results supported the higher cellular uptake of PEGylated PLGA NC in CT26 cells, due to PEG pronounced effect on decreasing nanoparticles size and minimising the electrostatic repulsion force with the cell membrane [12].

Given that SL and the other formulation components possess no fluorescent activity, it was necessary to add a fluorescent dye to determine the position of NC following exposure in the test cell lines. Images from fluorescence microscopy supported particulate uptake in CT26 cells (Fig. 5). The SL-loaded NC (F9) were primarily localised in the cytoplasm, while some fluorescence intensity was observed around the nucleus, which supports possible intracellular localisation of NC (Fig. 5g–i). Cells treated with coumarin 6 solution showed minimal uptake as the dye was localised at the outer cell surface (Fig. 5d–f). Based on these quantitative and qualitative cellular uptake results, it can be concluded that SL-loaded 10% PEGylated PLGA NC (F9) showed maximum intracellular uptake.

### In vitro cytotoxicity

An assessment of the physicochemical data for all formulations used in this work allowed selection of F9 as an optimised formulation. This was based on F9 having the smallest size, highest %EE, optimum shelf-life stability and highest cellular uptake. Therefore, F9 was selected for further cell viability and organ distribution studies.

**Fig. 6:**
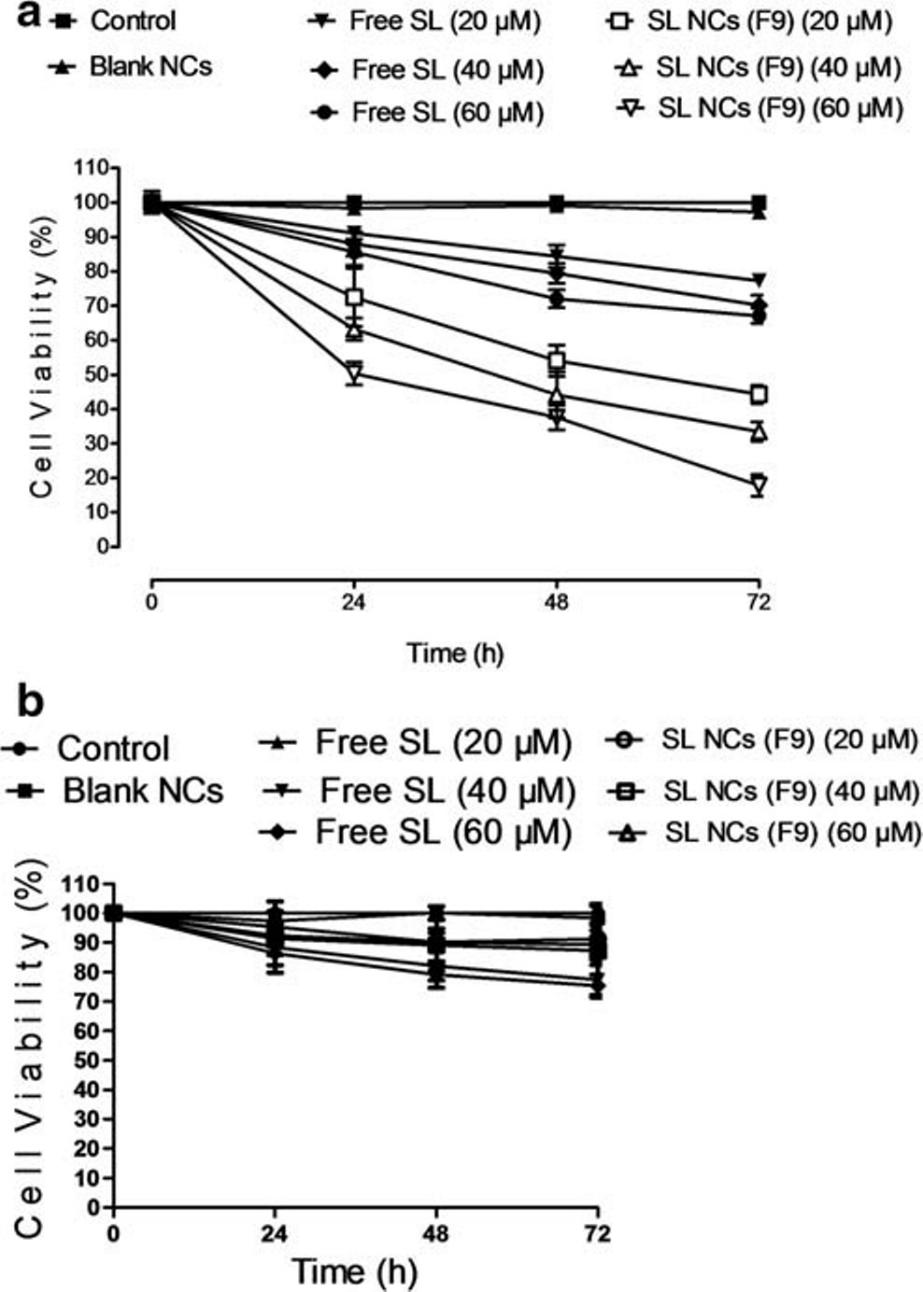
CT26 cell viability results of different doses of free SL and SL-loaded NC (F9) after 24, 48 and 72 h (**a**). CCD-841-CoN cell viability results of different doses of free SL and SL-loaded NC (F9) after 24, 48 and 72 h (**b**). Values are expressed as mean ± SD with *n =* 3

The cytotoxic action of SL-loaded NC (F9) on CT26 colon cancer cells and CCD-841-CoN normal colon epithelial cells was evaluated by MTT assay after 24, 48 and 72 h of treatment with 20, 40 and 60 μM of free SL and SL-loaded NC (Fig. 6a). Blank NC had no cytotoxicity action on colon cancer cells. The dose-response curves were generated to detect the IC_50_ which is the drug concentration resulting in 50% growth inhibition of colon cancer cells. Free SL showed a dose-dependent effect as cell viability decreased with increasing dose from 20 to 60 μM. Significant reduction in cell viability compared to control (*p <* 0.05) was observed. Treatment with SL-loaded NC (F9) reduced cell viability in a dose-dependent effect meanwhile it maintained its cytotoxic action up to 3 days after treatment due to sustained drug release. The mean IC_50_ value for SL-loaded NC (F9) in CT26 cells was approximately 60 μM, which was achieved within 24 h. Different doses of free SL showed a slight decrease in cell viability of CCD-841-CoN normal colon epithelial cells especially after 3 days of treatment (Fig. 6b). Twenty-five percent reduction of cell viability was observed after treatment with the highest dose of 60 μM of free SL after 72 h. On the other hand, SL-loaded NC (F9) achieved non-significant (*p ˃* 0.05) cytotoxic effect on normal colon epithelial cells within the concentration range used in this study. This confirms the safety profile of the designed drug delivery system compared to free drug for further in vivo applications. Interestingly, these results are different from other studies [10]. This might be due to the variation of purity of SL used, due to different sources also due to the lower range of drug concentration used in this study. These results showed that NC drug delivery system can deliver SL to its subcellular site and protect it from possible degradation to achieve lower cell viability and greater cytotoxicity when compared to the free SL after 24, 48, and 72 h of treatment (*p <* 0.001).

### In vivo tumour uptake and organ biodistribution study

**Fig. 7:**
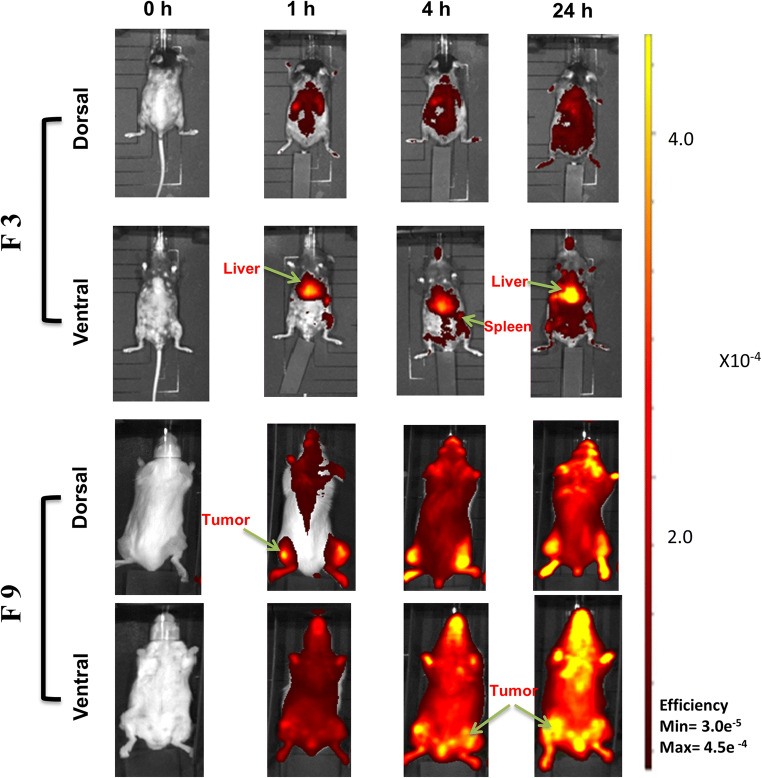
In vivo organ biodistribution of DiR-labelled SL-loaded NC (F3 and F9) in CT26 tumour-bearing Balb/c mice after i.v. injection. Representative whole body in vivo images obtained at 1, 4 and 24-h post-injection. All images were obtained by in vivo imaging system (IVIS Lumina III). Acquired data were analysed by Living Image 4.3.1 Service Pack 2 software

**Fig. 8:**
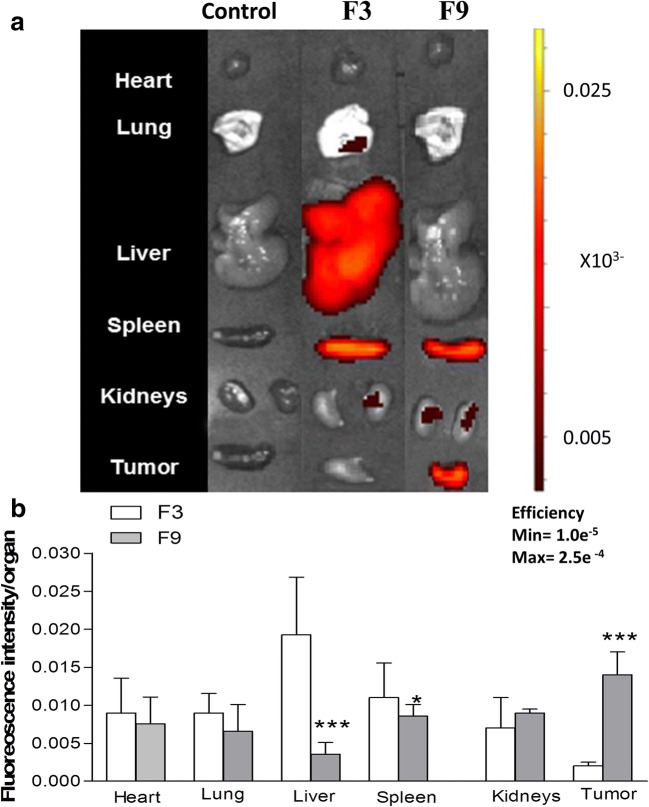
Ex vivo organ biodistribution of DiR-labelled SL-loaded NC (F3 and F9) in CT26 tumour-bearing Balb/c mice after IV injection. Representative ex vivo images of excised organs after 24 h of injection (**a**). All images were obtained by IVIS Lumina III. Acquired data were analysed by Living Image 4.3.1 Service Pack 2 software. Ex vivo quantification of fluorescence signals of SL-loaded NC (F3 and F9) per gram of excised organs and tumours after 24 h was represented (**b**). Values are presented as mean ± SD (*n =* 4), For 8**b**, *p < 0.05, *p <* 0.01, ***p < 0.001 compared with PLGA (F3)

Tumour accumulation and organ biodistribution of DiR-tagged SL-loaded NC (F3 and F9) were assessed in vivo in mice bearing the murine colon cancer CT26 model. Whole-body in vivo imaging was performed at 1, 4 and 24 h after injection (Fig. 7). Imaging of excised organs, such as the heart, lung, liver, spleen, kidneys and the tumour mass is shown in Fig. 8.

In vivo imaging data from mice treated with PBS (control group) confirmed that no background fluorescence was present (data not shown). SL-loaded NC (F3) formulated from PLGA polymer showed no tumour uptake after 1, 4 and 24 h, but did confirm accumulation in the liver and spleen (Fig. 7). Tumour uptake of 10% PEGylated NC (F9) was observed in both implanted tumours when imaged from the dorsal and ventral view.

The excised organs from control animals (treated with PBS) showed no fluorescence. Animal treated with F3 showed high fluorescence from both liver and spleen, but low fluorescence from the tumour masses. Excised organs from animals treated with F9 showed the highest fluorescence intensities from the tumours when compared to other organs (Fig. 8a). Fluorescence intensities from each organ were further quantified (Fig. 8b) showing mean fluorescence intensities from tumours treated with F9 to be highly significant compared to animals treated with F3 (*p <* 0.001) which confirms high tumour accumulation of F9. Fluorescence intensity quantified from the liver in animals treated with F3 was highly significant compared to animals treated with F9 (*p <* 0.001).

Size and surface properties of nanocarriers play an important role in controlling drug pharmacokinetics, biodistribution and drug resistance in some cancer cells [32, 33]. Nanoparticles ≥ 200 nm and without surface modification will induce the host immune systems to be engulfed by hepatic Kupffer cells and other reticuloendothelial cells, resulting in their rapid clearance from the circulation system. Nanoparticles with size ≤ 150 nm and PEG modification appear to be appropriate for passive targeting as they are better able to exploit site-specific endothelial irregularities and histological variability [28, 34, 35]. An appreciation of this often-debated EPR effect in solid tumours is one of the essential prerequisites for designing a successful antitumour drug delivery. As shown in this study, F9 fulfils the requirements for a long-circulating NC as its size (117.5 ± 11.5 nm) and PE-decorated surface are considered to be desirable features. F9 is, therefore, optimum for passive targeting to colon cancer due to its preferable biodistribution, which will lead to the highest therapeutic efficacy in vivo.

### In vivo antitumour activity

A low number of studies have investigated the therapeutic potential of SL in vivo. A sepsis model has been used, which showed that SL mixtures containing both acidic and lactonic SL reduced mortality in rats with experimentally induced sepsis via cecum puncture [36]. Recently, in vivo anticancer models have been used to study the anticancer activity of SL against human cervical cancer [11].

**Fig. 9:**
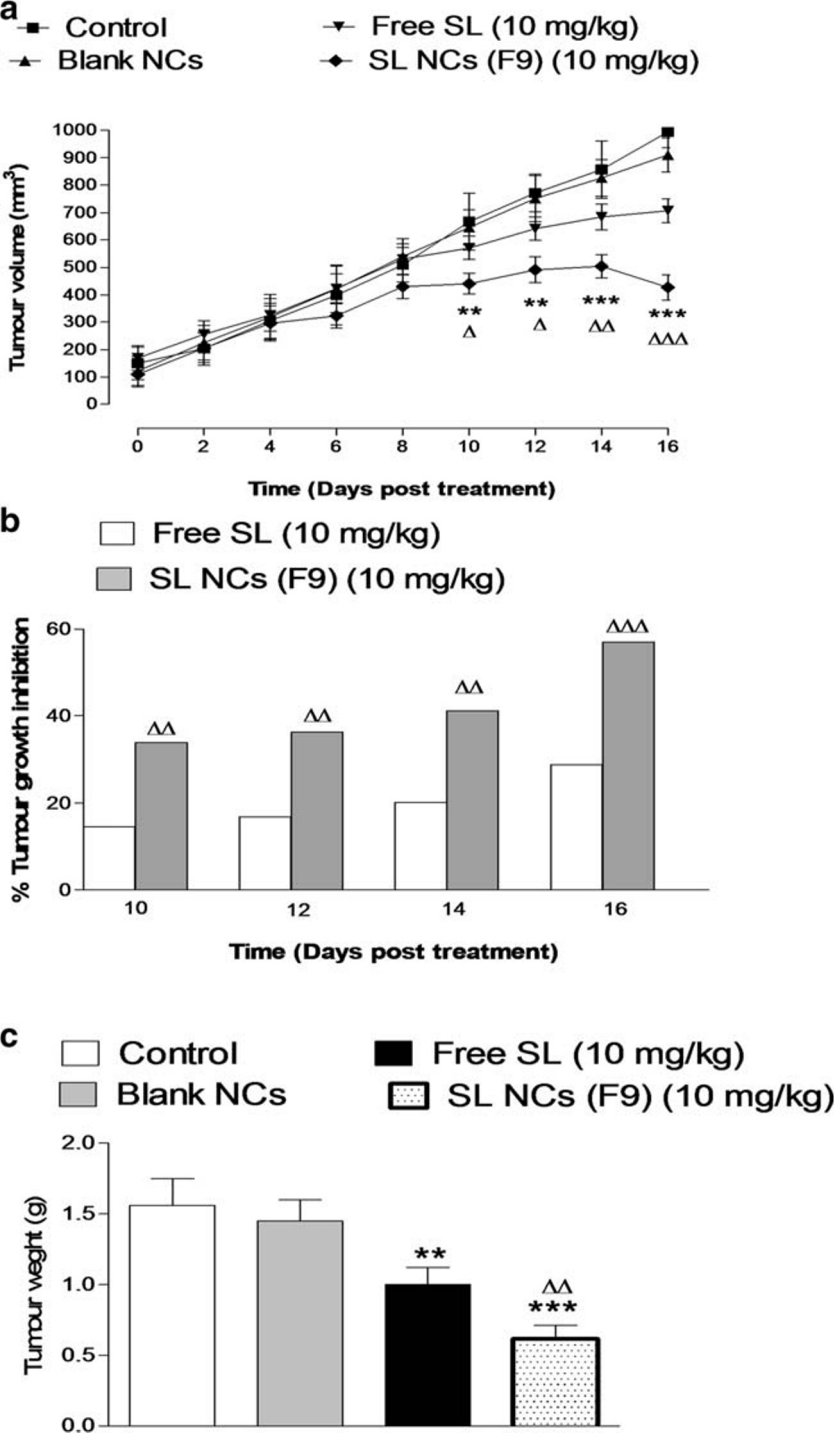
Tumour volume recorded for the studied animal groups every 2 days from the 1st day of the treatment to the last record at the 16th day (the end of the experiment). Values are mean ± SEM with *n =* 6. *p < 0.05, **p < 0.01, ***p < 0.001 compared with control group. ^Δ^*p <* 0.05, ^ΔΔ^*p <* 0.01, ^ΔΔΔ^*p <* 0.001 compared with animal treated by free SL at a dose of (10 mg kg^−1^) (**a**). Percentage tumour growth inhibition (% TGI) in animals treated with free SL or SL-loaded NC (F9) at a dose of (10 mg kg^−1^) ^Δ^*p* < 0.05, ^ΔΔ^*p* < 0.01, ^ΔΔΔ^*p* < 0.001 compared with animal treated by free SL (**b**). Tumour weight of studied groups after the end of the experiment. Values are mean ± SEM with *n =* 6. *p* *<* 0.05, **p < 0.01, ***p < 0.001 compared with control group. ^Δ^*p <* 0.05, ^ΔΔ^*p <* 0.01, ^ΔΔΔ^*p <* 0.001 compared with animals treated by free SL at a dose of (10 mg kg^−1^) (**c**)

All animal involved in this study appeared healthy throughout the whole experiment duration and no significant (*p ˃* 0.05) loss in body weight was detected. There were no toxicity signs after treatment with either free SL or SL-loaded NC (F9) at the drug concentration used. The dose used for this study was 10 mg SL per kg of mice weight, and 5 injections were administrated in vivo to the mice. The in vivo tumour growth inhibition study in mice bearing CT26 colon cancer after SL treatment showed no antitumour activity of blank NC and a pronounced anticancer effect of SL-loaded NC (F9) (Fig. 9a). The average tumour size of animals treated with F9 was significantly lower compared to the control group and animals treated with the free drug (*p <* 0.001). At the end of the experiment, the average size of the tumour mass of control animals treated with saline was found to be 993.5 ± 142.5 mm^3^. The average tumour size of animals treated with free SL was 706.7 ± 105.3 mm^3^ and the percentage tumour growth inhibition (% TGI) was 28.9%. A more pronounced effect was recorded in animals treated with F9. There was a significant reduction in the volume of tumour mass at each time point starting from the 10th day till the end of the experiment when compared with control and free drug-treated group. The final average size of tumour was found to be decreased to 426.6 ± 115.4 mm^3^ and the % TGI was 57.09% (Fig. 9b). It was observed that the F9 achieved a significant reduction in tumour size (*p <* 0.001) confirming its greater antitumour activity than free SL.

At the end of the in vivo experiment, all mice were sacrificed, and implanted tumours were excised and weighed (Fig. 9c). The change in tumour weights was observed in the case of animals treated with free SL or F9. Treatment of mice with F9 reduced the average tumour weight to 0.62 ± 0.1 g compared to 1.6 ± 0.46 g for control animals with a percentage reduction of 61.25%, which is highly significant compared to control (*p <* 0.001) and free drug-treated mice (*p <* 0.01). The percentage reduction in tumour weights was 37.5% in mice treated with free SL, which is statically significant compared to control mice (*p <* 0.01).

## Conclusions

This study investigated, for the first time, the formulation and optimisation of a nanocapsule drug delivery system for SL encapsulation and in vivo administration in CT26 colon cancer. Formulation of PEG-PLGA-based polymeric NC with an oil core showed high SL loading and sub-micrometre particle size. PEGylated 10% NC (F9) showed superior physio-chemical properties compared to similar SL-loaded PLGA NC and a favourable shelf-life stability. This formulation achieved efficient cellular uptake by CT26 colon cancer cell line compared to other polymeric NC which facilitated its cytotoxic action in vitro meanwhile it was safe to normal colon cells. PEGylation makes F9 suitable for passive targeting due to a prolonged blood circulation profile by evading the immune system thus maximising its accumulation in colon tumour mass and minimising its unnecessary biodistribution to other body organs after i.v. administration. F9 was capable of inhibition of tumour growth and tumour weight in vivo while being safe to treated subjects. In conclusion, these results suggest that SL-loaded 10% PEGylated NC could be an effective anticancer therapy for colon cancer. Our future work will involve testing the anticancer activity of this novel drug delivery system against different types of tumours.
